# Maackiain Ameliorates 6-Hydroxydopamine and *SNCA* Pathologies by Modulating the PINK1/Parkin Pathway in Models of Parkinson’s Disease in *Caenorhabditis elegans* and the SH-SY5Y Cell Line

**DOI:** 10.3390/ijms21124455

**Published:** 2020-06-23

**Authors:** Rong-Tzong Tsai, Chia-Wen Tsai, Shih-Ping Liu, Jia-Xin Gao, Yun-Hua Kuo, Pei-Min Chao, Huey-Shan Hung, Woei-Cherng Shyu, Shinn-Zong Lin, Ru-Huei Fu

**Affiliations:** 1 Institute of Biochemistry, Microbiology and Immunology, Chung Shan Medical University, Taichung 40201, Taiwan; rttsai@csmu.edu.tw; 2 Department of Nutrition, China Medical University, Taichung 40402, Taiwan; cwtsai@mail.cmu.edu.tw (C.-W.T.); pmchao@mail.cmu.edu.tw (P.-M.C.); 3 Graduate Institute of Biomedical Sciences, China Medical University, Taichung 40402, Taiwan; spliu@mail.cmu.edu.tw (S.-P.L.); c1181130@gmail.com (J.-X.G.); hunghs@mail.cmu.edu.tw (H.-S.H.); shyu9423@gmail.com (W.-C.S.); 4 Translational Medicine Research Center, China Medical University Hospital, Taichung 40447, Taiwan; 5 Department of Nursing, Taipei Veterans General Hospital, Taipei 12217, Taiwan; yhkuo3@vghtpe.gov.tw; 6 Department of Neurosurgery, Bioinnovation Center, Tzu Chi foundation, Buddhist Tzu Chi General Hospital, Tzu Chi University, Hualien 970, Taiwan; shinnzong@yahoo.com.tw; 7 Department of Psychology, Asia University, Taichung 41354, Taiwan

**Keywords:** Parkinson’s disease, maackiain, 6-Hydroxydopamine, α-Synuclein, *Caenorhabditis elegans*, SH-SY5Y cell, apoptosis, autophagy, proteasome, parkin

## Abstract

The movement disorder Parkinson’s disease (PD) is the second most frequently diagnosed neurodegenerative disease, and is associated with aging, the environment, and genetic factors. The intracellular aggregation of α-synuclein and the loss of dopaminergic neurons in the substantia nigra pars compacta are the pathological hallmark of PD. At present, there is no successful treatment for PD. Maackiain (MK) is a flavonoid extracted from dried roots of *Sophora flavescens* Aiton. MK has emerged as a novel agent for PD treatment that acts by inhibiting monoamine oxidase B. In this study, we assessed the neuroprotective potential of MK in *Caenorhabditis elegans* and investigated possible mechanism of this neuroprotection in the human SH-SY5Y cell line. We found that MK significantly reduced dopaminergic neuron damage in 6-hydroxydopamine (6-OHDA)-exposed worms of the BZ555 strain, with corresponding improvements in food-sensing behavior and life-span. In transgenic worms of strain NL5901 treated with 0.25 mM MK, the accumulation of α-synuclein was diminished by 27% (*p* < 0.01) compared with that in untreated worms. Moreover, in worms and the SH-SY5Y cell line, we confirmed that the mechanism of MK-mediated protection against PD pathology may include blocking apoptosis, enhancing the ubiquitin-proteasome system, and augmenting autophagy by increasing *PINK1/parkin* expression. The use of small interfering RNA to downregulate parkin expression in vivo and in vitro could reverse the benefits of MK in PD models. MK may have considerable therapeutic applications in PD.

## 1. Introduction

Parkinson’s disease (PD) is an aging-associated neurodegenerative movement disorder that is characterized by the progressive aggregation of α-synuclein in surviving neurons (named Lewy bodies) and the selective death of dopaminergic neurons in the substantia nigra pars compacta [1]. Symptoms include tremor at rest, rigidity, and slowed movements [2]. Studies show that by 2020, approximately 930,000 people in the United States will have the disease, and by 2030 the incidence is predicted to increase to 1.2 million [3].

Even though the precise triggers of PD remain uncertain, the interconnection of oxidative stress, mitochondrial impairment, protein misprocessing, and genetic variation plays a crucial role in the pathogenesis of the disease [4]. The movement of α-synuclein from the gut to the brain has also been proposed to be pathogenic [5], and some studies have shown that gut microbes are associated with the regulation of motor deficits in PD models [6]. Currently, there is no successful pharmacologic treatment for curing PD.

Reactive oxygen species (ROS), which are predominantly produced by mitochondria during oxidative stress, injury mitochondria and initiate apoptosis [7]. 6-Hydroxydopamine (6-OHDA) is a neurotoxic compound widely used in various PD models to selectively destroy dopaminergic neurons by the production of ROS via dopaminergic reuptake transporters [8]. Some studies have shown that treatment with 6-OHDA can induce apoptosis, reduce proteasome activity, and block autophagy in cell models [9,10].

α-Synuclein (a product of the *SNCA* gene) is found mainly at presynaptic terminals of neurons. Studies indicate that α-synuclein plays a key role in restricting the mobility of synaptic vesicles, lessening neurotransmitter release and synaptic vesicle recycling [11]. Abnormal aggregates (inclusions and aggresomes) of wild-type or mutated α-synuclein may block the cellular functions associated with the degradation system, mitochondria, and chaperone proteins, resulting in a rapid loss of whole-cell homeostasis. For instance, Snyder et al. indicated that α-synuclein reduces proteasomal activity by binding to the Rpt3 of the 19S AAA-ATPase subunit [12]. Thereby, α-synuclein aggregates are toxic in various PD models and lead to neuronal degeneration [13].

Studies have revealed that the PINK1/parkin pathway protects cells from various types of cellular stress through the ubiquitin-proteasome system (UPS), mitochondrial quality control mechanisms, and autophagy [14]. PINK1 (PTEN-induced kinase 1) is a serine/threonine kinase found in the inner membrane of mitochondria. Parkin is a ubiquitin E3 ligase that promotes protein degradation by the 26S proteasome via the addition of ubiquitin on target proteins. PINK1 accumulates on the outer membrane of depolarized mitochondria, then recruits and activates parkin via phosphorylation of the ubiquitin chains, and finally induces damaged mitochondria to degrade by autophagy and the UPS [15]. Autophagy assists in the degradation of long-lived and abnormally aggregated proteins as well as injured organelles. The process of removing damaged mitochondria by autophagy is called mitophagy. A defect of the PINK1/parkin pathway results in mitophagy dysfunction [15]. Knockout of PINK1 and parkin in the mouse midbrain reduces mitochondrial function, quantity, and the survival of dopaminergic neurons [16]. Parkin-mediated lysine 48-linked ubiquitination is commonly associated with proteasome degradation [17]. However, lysine 63- or 27-linked ubiquitinated proteins are involved in autophagy [18]. In addition, parkin works synergistically with the autophagy protein Beclin1 in autophagosome maturation [19].

Parkin can also interact with the Rpn 1, Rpn 10, and Rpt 5 subunits of the 19S proteasome and the α 4 subunit of the 20S proteasome by a ubiquitin-like domain to activate the 26S proteasome [20]. One analysis showed that PD patients with a *parkin* mutation in the substantia nigra region have lower proteasome activity [21]. In *Drosophila melanogaster* and mice, Parkin knockout diminishes 26S proteasome activity, whereas overexpression increases its activity [21]. In addition, parkin increases cell survival by inhibiting both mitochondria-dependent and mitochondria-independent apoptosis [22]. VDAC1 is one of parkin’s substrates and is responsible for regulating apoptosis and mitophagy. VDAC1 lacking PINK1/parkin-dependent monoubiquitination stimulates apoptosis, but VDAC1 lacking polyubiquitination inhibits mitophagy [23].

Sequence variations in PINK1 and parkin mutations are known to be associated with autosomal recessive parkinsonism in some patients with PD [24]. Recent research indicates that PINK1 is a repressor of PD-associated autoimmune events. For example, intestinal infection in *Pink1*^−/−^ mice was shown to involve antigen presentation of mitochondrial, causing the development of cytotoxic mitochondria-specific CD8^+^ T cells and triggering PD [25].

**Figure 1:**
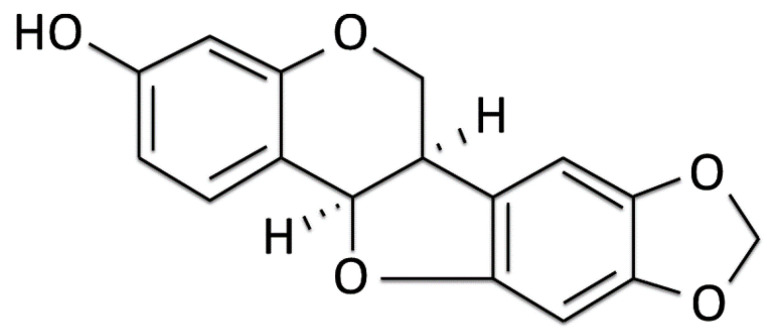
Chemical structure of maackiain.

*Sophora flavescens* is a traditional Chinese herbal medicine, namely Kushen [26]. Maackiain (MK, Figure 1) has been isolated from the dried roots of *Sophora flavescens* Aiton. and has been reported to have multiple pharmacologic properties, such as the inhibitory activity on monoamine oxidase B [27] and anti-allergic [28], anti-cancer [29], and anti-inflammatory activities [30]. However, the effectiveness of MK against PD has not been evaluated. The nematode *Caenorhabditis elegans* is a dominant model organism in which to study PD and can be used as a simple drug-screening platform [31,32,33]. Here, we used *C. elegans* and human SH-SY5Y cell models to assess the anti-Parkinsonian effects of MK and to address its potential neuroprotection mechanisms in vivo and in vitro.

## 2. Results

### 2.1. Using the Food Clearance Test to Determine the Concentration Range of Maackiain Treatment in C. Elegans

**Figure 2:**
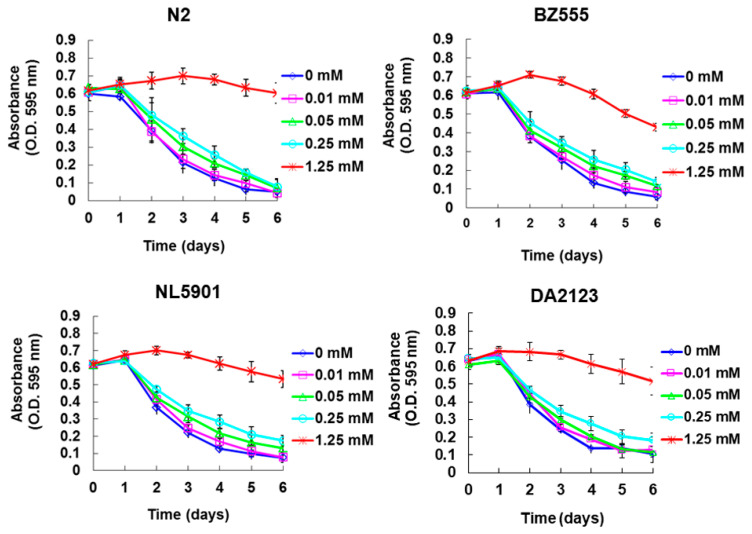
The concentration of maackiain (MK) used in the *C. elegans* model was determined by a food clearance test. In a 96-well plate, synchronized L1 worms of N2, BZ555, NL5901, and DA2123 strains were cultured with *E. coli* in OP50 (OD A_595_ = 0.6) feeding medium containing 0, 0.01, 0.05, 0.25 or 1.25 mM MK for 6 days. During this period, the OD value of each treatment was measured and recorded daily.

We used a food clearance test to determine the suitable concentration of MK for use in the *C. elegans* PD model. Addition of 0.01, 0.05, or 0.25 mM MK to the cultures of N2, BZ555, NL5901, or DA2123 strains did not significantly affect the curves of food clearance compared with that in control worms. However, worms exposed to 1.25 mM MK had significantly reduced food clearance (Figure 2). In addition, worms exposed to the higher concentration of 1.25 mM MK had fewer numbers of offspring and reduced body sizes (data not shown), which are linked to a deficiency of *E. coli* clearance. Therefore, in subsequent assays, the concentration of MK used in the treatment of worms was up to 0.25 mM.

### 2.2. Maackiain Diminished Dopaminergic Neuron Degeneration Caused by 6-OHDA Exposure in Worms

**Figure 3:**
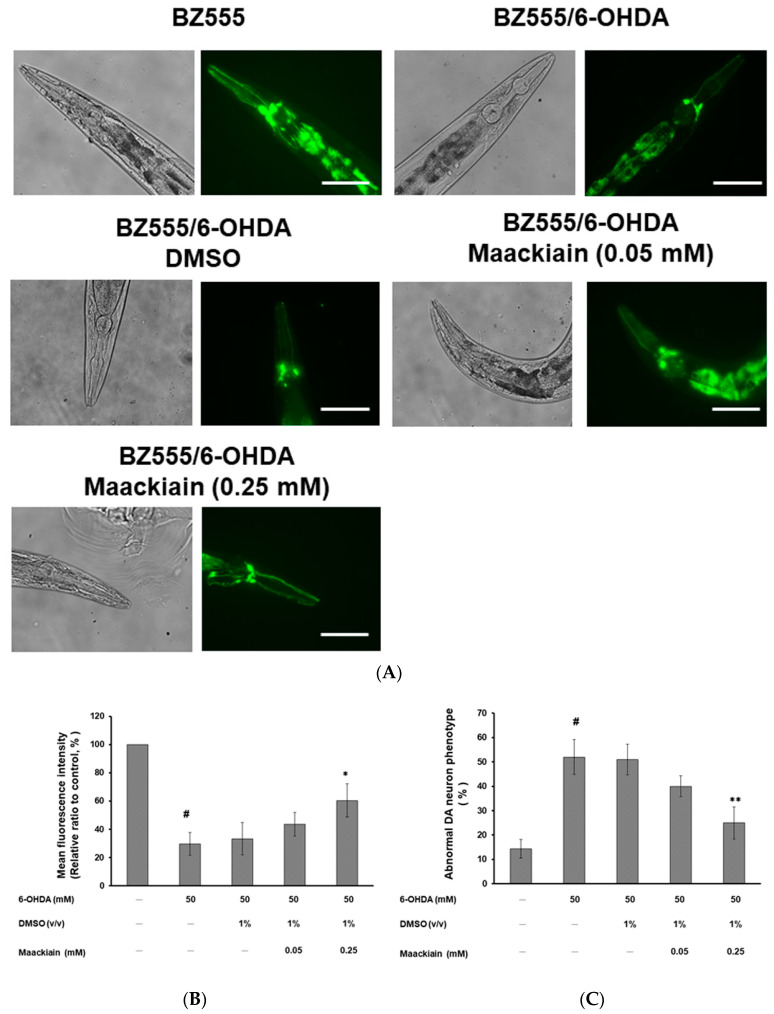

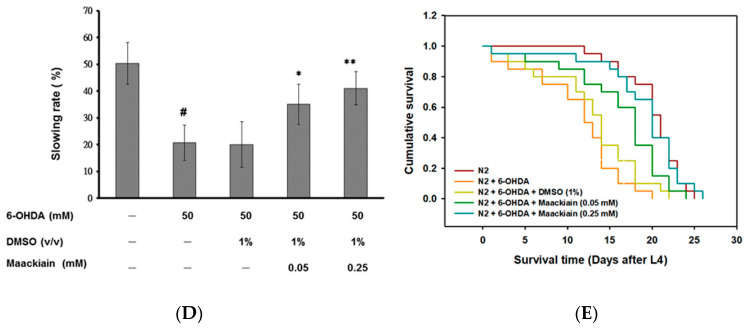
6-hydroxydopamine (6-OHDA)-induced degeneration of dopaminergic (DA) neurons, defects in food-sensing behavior, and shortening of life-span in the BZ555 strain were ameliorated by maackiain (MK) treatment. MK-pretreated or untreated L3 stage BZ555 worms were exposed to 6-OHDA and then cultured for an additional 3 days. (**A**) Representative fluorescence images of GFP expression in DA neurons. Scale bar = 50 µm. (**B**) Quantifying of GFP fluorescence intensity pattern in 50 animals per group using ImageJ software. (**C**) Degenerative phenotypic defects of DA neurons were scored for 50 animals per group. Data are presented as a percentage of the total population with abnormal phenotypes in each treatment group. (**D**) Slowing rates were calculated as the percentage lessening in frequency of body bending (20 s) in the bacterial lawn compared to without a bacterial lawn for 50 animals per group. (**E**) Cumulative survival curves for worms cultured in OP50/NGM plates until all worms died (50 animals per group). In the above experiments, # shows significant differences between 6-OHDA-exposed and control worms (# *p* < 0.001); * shows significant differences between the MK-pretreated 6-OHDA-exposed and MK-untreated 6-OHDA-exposed groups (* *p* < 0.05, ** *p* < 0.01).

On day three after synchronized L3 worms of strain BZ555 were exposed to 6-OHDA treatment, we found that GFP expression in dopaminergic neurons in the head (ADE and CEP) was partially reduced (Figure 3A). MK-pretreated 6-OHDA-exposed worms showed recovery of the GFP signal (Figure 3A). We further used ImageJ software to quantify the fluorescence intensity in dopaminergic neurons. Compared with that in unexposed worms, the average fluorescence intensity (GFP) was lessened by 70% (*p* < 0.001) in 6-OHDA-exposed worms (Figure 3B). Moreover, MK dose-dependently elevated the fluorescence intensity of GFP. Compared with that in worms treated with 6-OHDA alone, the fluorescence intensity in dopaminergic neurons in 6-OHDA-exposed worms was increased 1.8-fold (*p* < 0.05) by treatment with 0.25 mM MK (Figure 3B).

In addition, compared with that in the unexposed control group, exposure to 6-OHDA significantly augmented the percentage of abnormal phenotypes by 3.2-fold (*p* < 0.001) (Figure 3C). After MK (0.25 mM) was added, 6-OHDA-exposed worms showed a significant 52% reduction (*p* < 0.01) in phenotypes of neuronal degeneration (Figure 3C).

### 2.3. Recovery of Food-Sensing Behavior by Maackiain Treatment in 6-OHDA-Exposed Worms

Our results showed that the bending frequency of wild-type N2 worms was reduced by 50.1% after contact with a bacterial lawn (Figure 3D). We next tested whether worms exposed to 6-OHDA exhibited defect in food-sensing behavior (quantified as the “slowing rate”). Three days after 6-OHDA exposure, worms showed a significant lessening in slowing rate compared with wild-type N2 worms (21%, *p* < 0.001). MK dose-dependently recovered the slowing rate in 6-OHDA-exposed worms. Compared with worms treated with 6-OHDA alone, in worms also treated with 0.25 mM MK, the bending movements of worms decreased 2-fold after contact with a bacterial lawn (*p* < 0.01) (Figure 3D).

### 2.4. Maackiain Treatment Augments Life-Span of 6-OHDA-Exposed Worms

Compared with that of wild-type N2 worms, 6-OHDA-exposed worms was shorter (Figure 3E). MK pretreatment extended the lifespans of 6-OHDA-exposed worms in a dose-dependent manner. Figure 3E shows the cumulative survival patterns of longevity estimated by using the Kaplan–Meier method for each experiment. The mean survival time for the 6-OHDA-exposed group was 14.4 ± 1.11 days vs. 19.1 ± 2.24 days for the MK-pretreated (0.25 mM) 6-OHDA-exposed group (*p* < 0.001).

### 2.5. α-Synuclein Protein Accumulation Was Diminished by Maackiain Treatment

**Figure 4:**
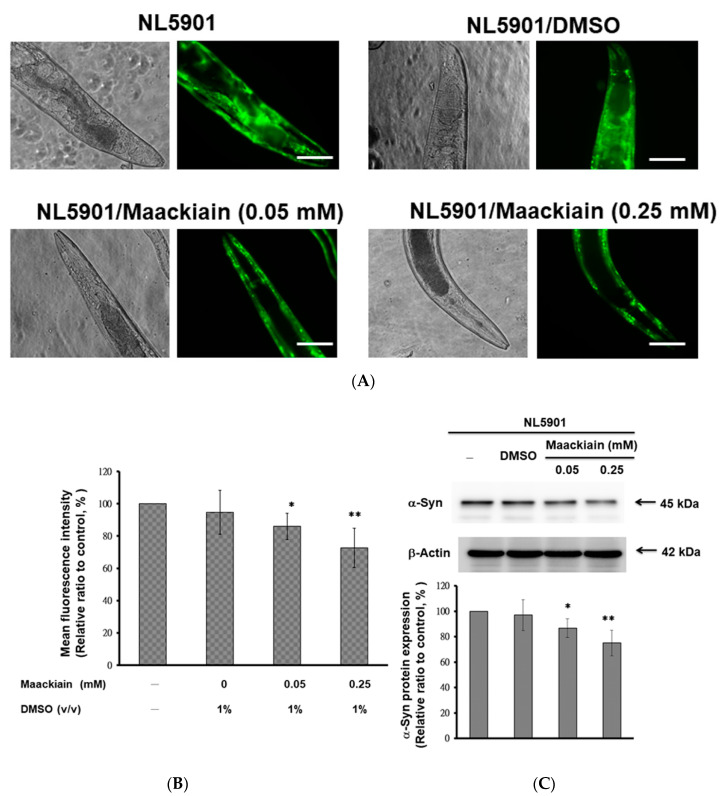
Accumulation of α-synuclein (α-Syn) was diminished by maackiain (MK) treatment in the NL5901 strain of *C. elegans*. L3 stage worms were treated with or without MK and cultured until the third day of adulthood. (**A**) The representative YFP fluorescence images of α-Syn accumulation in the muscles of the head region of worms. Scale bar = 50 µm. (**B**) ImageJ software was used to quantify YFP fluorescence intensity for 50 animals per group. (**C**) The protein level of α-Syn in the MK-treated and MK-untreated worms was determined by Western blotting. A representative result from one of three independent experiments is shown. The level of β-actin was used as an internal control for loading. The relative fold in α-Syn level is represented as the ratio of the MK-treated groups relative to the MK-untreated groups. In the above experiments, * shows significant differences between the MK-untreated and the MK-treated worms (* *p* < 0.05, ** *p* < 0.01).

MK dose-dependently reduced the fluorescence intensity of YFP, which was associated with α-synuclein accumulation in NL5901 worms (Figure 4A). In worms treated with 0.25 mM MK, the fluorescence intensity was weakened by 27% (*p* < 0.01) compared with that in untreated worms (Figure 4B).

We also wanted to clarify the cause for the decrease in α-synuclein fluorescence intensity in MK-treated NL5901 worms by Western blotting. The results showed that MK did not lessen the aggregation of α-synuclein (data not shown), but did diminish its accumulation. NL5901 worms treated with MK displayed dose-dependently reduced protein levels of α-synuclein (Figure 4C). With 0.25 mM MK treatment, α-synuclein levels of NL5901 worms were decreased by 25.1% compared with those in untreated worms (*p* < 0.01) (Figure 4C).

### 2.6. Reduction in Dopaminergic Neuron Degeneration in 6-OHDA-Exposed Worm Model by Maackiain Treatment Linked to Reactive Oxygen Species Level and Expression of Pink1 and Pdr-1

**Figure 5:**
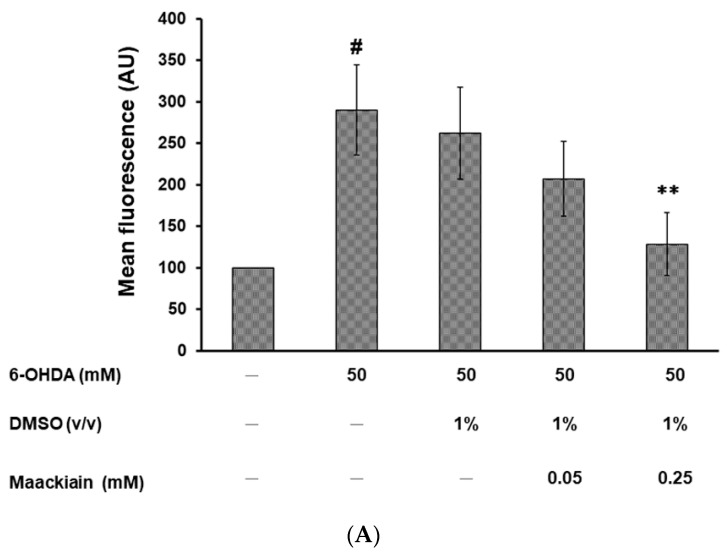

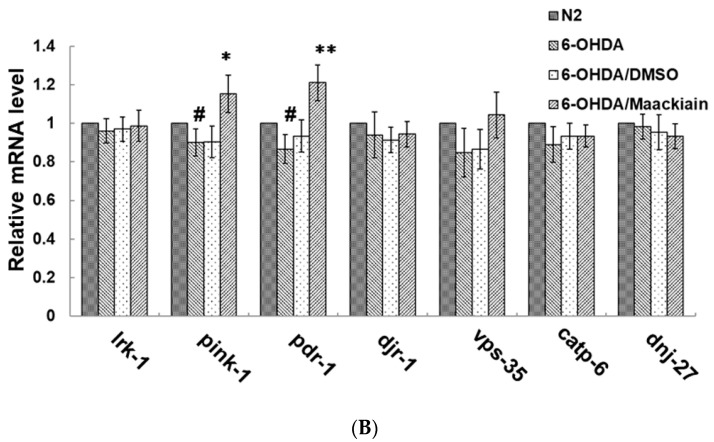
Maackiain (MK) significantly diminished the intracellular reactive oxygen species (ROS) level and increased *pink1* and *pdr-1* expression in 6-hydroxydopamine (6-OHDA)-exposed N2 *C. elegans*. MK-pretreated or untreated L3 stage worms were exposed to 6-OHDA for 1 h and were then cultured in the NGM plate for 3 days. (**A**) Thirty randomly selected worms from each experimental group were transferred to the well of a 96-well plate. The intracellular ROS level was evaluated. # shows significant differences between 6-OHDA-exposed and control worms (# *p* < 0.01); * shows significant differences between the MK-untreated 6-OHDA-exposed worms and MK-pretreated 6-OHDA-exposed worms (** *p* < 0.01). (**B**) The expression level of PD-associated genes in *C. elegans* was quantified by qPCR. # shows significant differences between 6-OHDA-exposed and control worms (# *p* < 0.05); * shows significant differences between the MK-untreated 6-OHDA-exposed worms and MK-pretreated 6-OHDA-exposed worms (* *p* < 0.05, ** *p* < 0.01).

We first wondered whether MK can reduce the levels of ROS in 6-OHDA-exposed N2 animals to improve the degeneration of dopaminergic neurons. Three days after 6-OHDA exposure, ROS levels were significantly increased by about 2.9-fold compared with those in the unexposed worms (*p* < 0.01). MK dose-dependently diminished the ROS levels of 6-OHDA-exposed worms. After pretreatment with 0.25 mM MK, the ROS levels of 6-OHDA-exposed worms were reduced by about 56% (*p* < 0.01) compared with levels in worms treated with 6-OHDA alone (Figure 5A).

Next, we used real-time PCR (qPCR) to detect the gene expression of *lrk-1*/*LRRK2*, *pink-1/PINK1*, *pdr-1/PREN/parkin*, *djr1.1/djr1.2/DJ-1*, *vps-35/VPS35*, *catp6/ATP13A2*, and *dnj-27*/*DNAJC10/Hsp40*, which are associated with the pathophysiology of PD in *C. elegans*. The experimental results showed the expression of *lrk-1*, *djr-1.1*/*djr-1.2*, *vps-35*, *catp6*, and *dnj-27* did not significantly differ in 6-OHDA-exposed worms and unexposed worms (Figure 5B), but the expression of *pink-1* and *pdr-1* decreased slightly in 6-OHDA-exposed worms (*p* < 0.05). With 0.25 mM MK treatment, the mRNA level of *pink-1* and *pdr-1* in 6-OHDA-exposed worms was increased by 28% (*p* < 0.05) and 40% (*p* < 0.01), respectively, compared the levels in worms treated with 6-OHDA alone (Figure 5B).

### 2.7. Maackiain Lessened α-Synuclein Accumulation through Pdr-1 (Parkin) Expression to Enhance Somatic Proteasome Activity and Autophagy 

**Figure 6:**
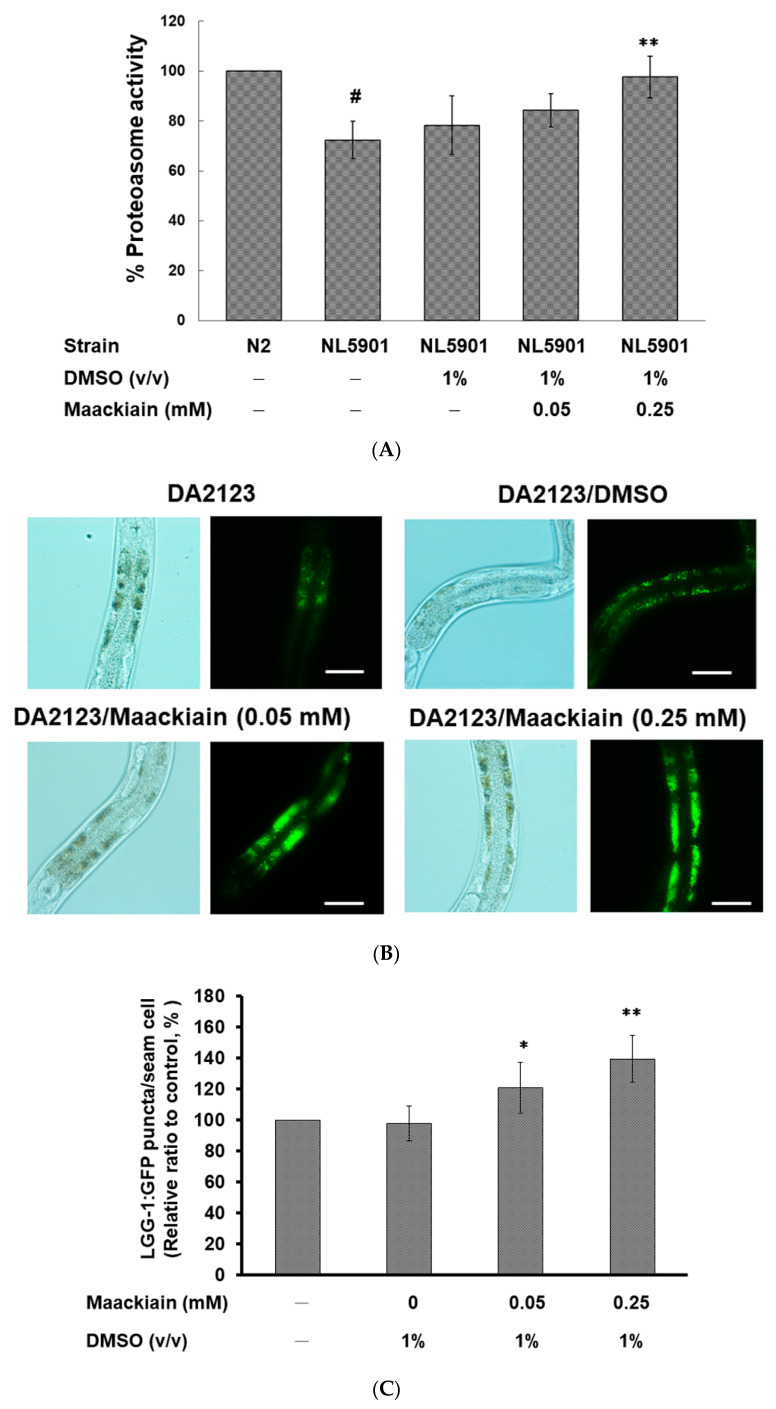

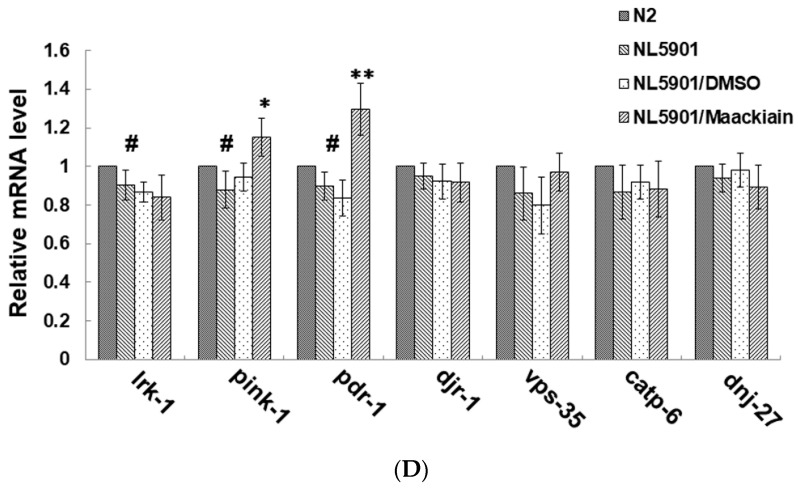
Maackiain (MK) treatment enhanced proteasome and autophagy activity and increased *pdr-1* expression in the transgenic *C. elegans* models. L3 stage worms of the NL5901 or DA2123 strain were treated with or without MK and cultured until the third day of adulthood. (**A**) The activity of the proteasome was monitored in the extract of NL5901 worms from different groups containing equal amounts of total protein. # shows significant differences between N2 and NL5901 worms (*p* < 0.01); * shows significant differences between the MK-untreated and MK-treated worms (** *p* < 0.01). (**B**) Representative GFP fluorescence images of positive puncta in lateral hypodermal seam cells of DA2123 worms are displayed. Scale bar = 10 µm. (**C**) The number of positive puncta was counted in the lateral epidermal seam cell of DA2123 worms. At least 50 worms were counted in each experimental group, and at least 100 seam cells were counted for each worm. * shows significant differences between MK-untreated and MK-treated worms (* *p* < 0.05, ** *p* < 0.01). (**D**) The expression level of the PD-associated genes was quantified by qPCR in NL5901 worms. # shows significant differences between N2 and NL5901 worms (*p* < 0.05); * shows significant differences between the MK-untreated and MK-treated NL5901 worms (* *p* < 0.05, ** *p* < 0.01).

Moreover, we wanted to confirm whether the reversal of α-synuclein accumulation with MK treatment was related to the activity of proteasome and autophagy in the *C. elegans* model. First, we assessed the effects of MK on the UPS in the NL5901 strain using a fluorogenic peptide substrate-based proteasome activity assay. The results showed that the basal level of proteasome activity was 28% lower in NL5901 worms than in N2 worms (*p* < 0.01) (Figure 6A). MK treatment dose-dependently augmented the proteasome activity in NL5901 worms. At 0.25 mM MK treatment, the proteasome activity of the worms was raised by 1.4-fold (*p* < 0.01) compared with that in the untreated group (Figure 6A).

We also used the transgenic strain DA2123 expressing the GFP-tagged LGG-1 to detect the activity of autophagy by counting the number of puncta in *C. elegans* seam cells (Figure 6B). Based on the experimental results, we found that the number of LGG-1::GFP puncta was augmented by 1.4-fold (*p* < 0.01) by treatment with 0.25 mM MK compared with that in untreated worms (Figure 6C).

Furthermore, we determined the mRNA expression levels of PD-associated genes in the NL5901 strain by using qPCR. The basal level of these genes was not significantly different in NL5901 worms than in N2 worms, with the exception of *lrk-1*, *pink-1*, and *pdr-1*, which were slightly decreased (*p* < 0.05) (Figure 6D). At 0.25 mM MK treatment, expression of *pdr-1* was significantly elevated 1.4-fold (*p* < 0.01) in the adult NL5901 worms (Figure 6D). MK also slightly increased expression of *pink-1* (*p* < 0.05) (Figure 6D). 

### 2.8. The Ability of Maackiain to Improve PD Pathology Can Be Abolished by Downregulating the Expression of Pdr-1

**Figure 7:**
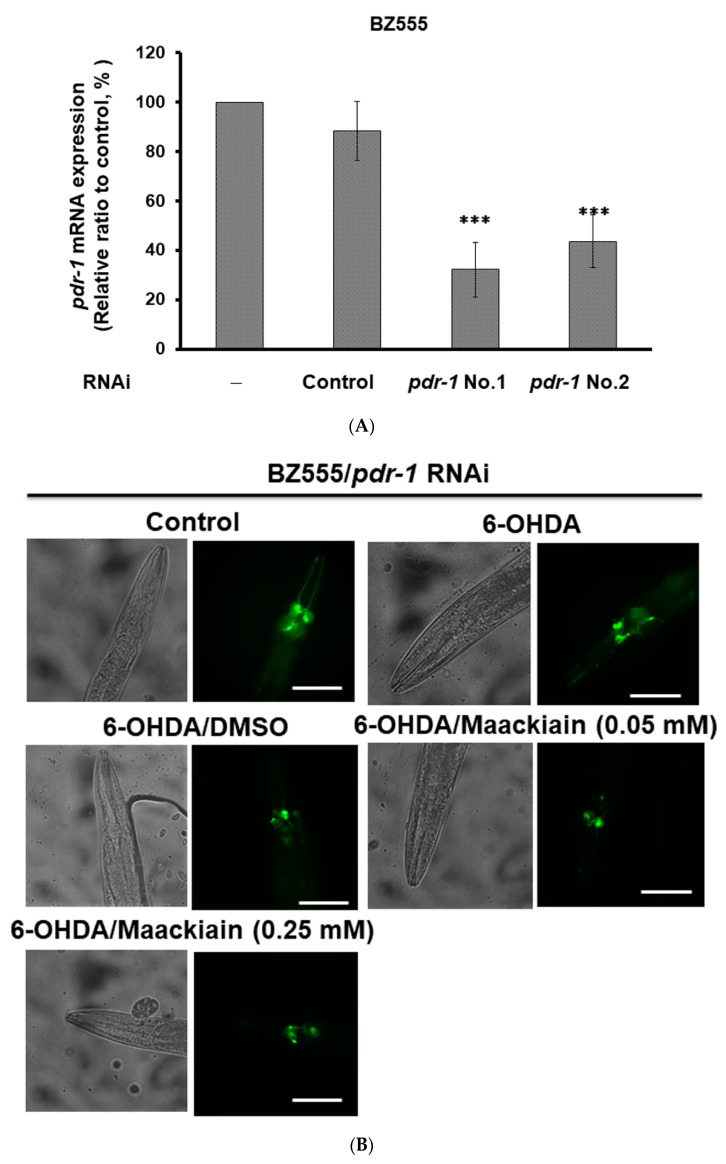

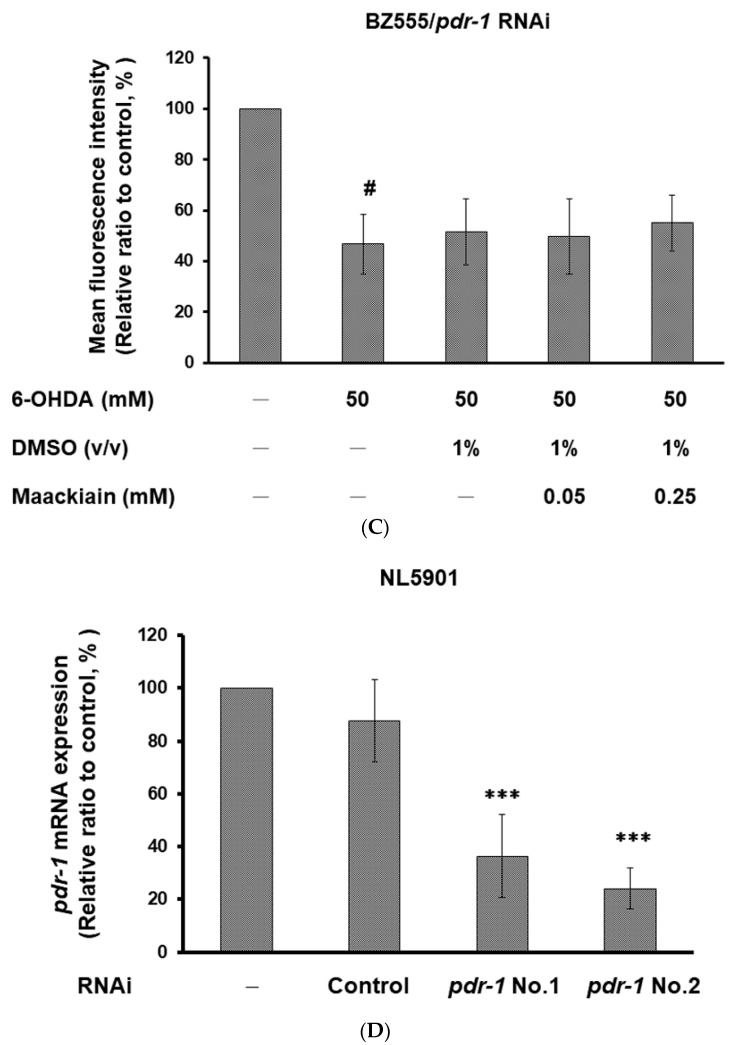

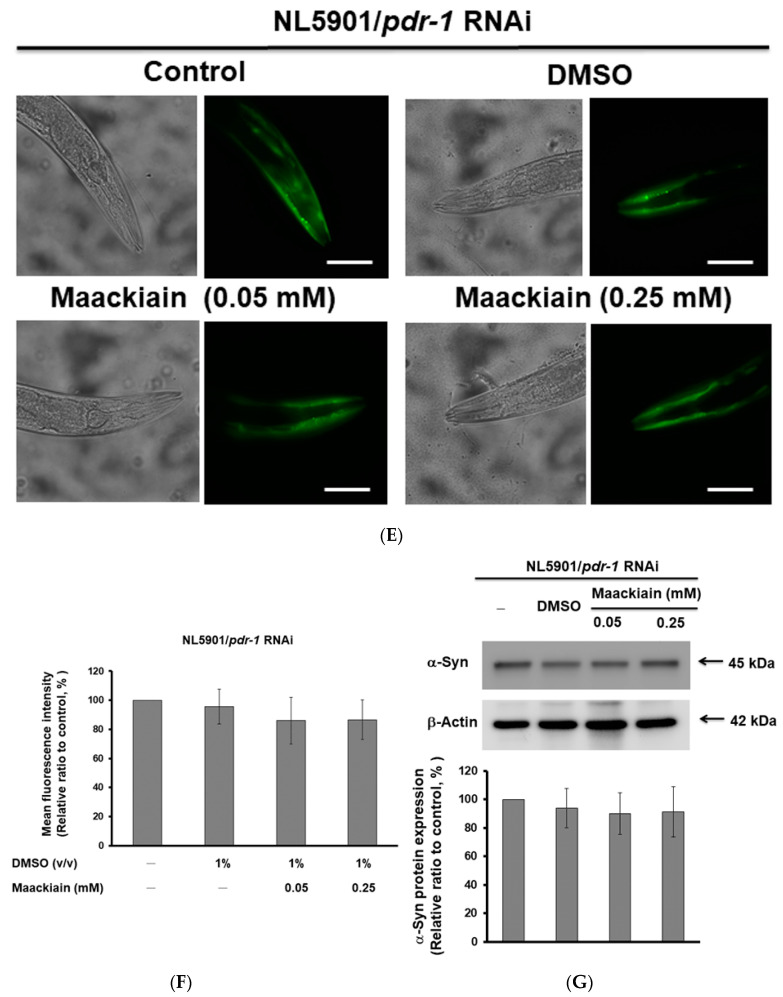
Using RNA interference (RNAi) approaches to downregulate the expression of *pdr-1* lessened the ability of maackiain (MK) to ameliorate the pathology of Parkinson’s disease (PD) in *C. elegans* models. (**A**) *Pdr-1* RNAi of BZ555 worms was performed by the method of feeding with bacteria expressing dsRNA. The mRNA level of *pdr-1* was measured by qPCR. The expression of β-actin was used as an internal control. * shows significant differences between the control RNAi-treated and *prd-1* RNAi-treated worms (*** *p* < 0.001). (**B**) Representative GFP fluorescence images of DA neurons are shown from different experimental groups. Scale bar = 50 µm. (**C**) ImageJ software was used to quantify the GFP fluorescence intensity of DA neurons from different groups. Comparisons are between the MK-untreated 6-OHDA-exposed and MK-pretreated 6-OHDA-exposed group. # shows significant differences between 6-OHDA-exposed and control worms (# *p* < 0.001) (**D**) *Pdr-1* RNAi of NL5901 worms was performed by the method of feeding with bacteria expressing dsRNA. The mRNA level of *pdr-1* was evaluated by qPCR. The expression of β-actin was used as an internal control. * shows significant differences between the control RNAi and *pdr-1* RNAi-treated worms (*** *p* < 0.001). (**E**) Representative YFP fluorescence images of α-synuclein (α-Syn) accumulation of muscle cells in the head region are shown from different groups of the NL5901 strain. Scale bar = 50 µm. (**F**) ImageJ software was used to quantify the YFP fluorescence intensity from different groups of the NL5901 strain. Comparisons are between the MK-untreated and MK-treated worms. (**G**) Western blotting analysis was used to quantify the protein level of α-Syn from different groups of the NL5901 strain. One representative result is shown. The expression of β-actin was used as an internal control. The relative fold protein level is represented as the ratio of the MK-treated worms relative to the MK-untreated worms.

Furthermore, we wanted to verify the role of *pdr-1* in MK-pretreated 6-OHDA-exposed BZ555 worms as well as in MK-treated NL5901 worms by using RNAi. Compared with the worms in the control RNAi group, RNAi reduced the expression of *pdr-1* by 68% in BZ555 worms (No. 1, *p* < 0.001) (Figure 7A). After 6-OHDA exposure, *pdr-1*-downregulated MK-pretreated BZ555 worms did not display enhanced GFP fluorescence intensity related to intact dopaminergic neurons compared with *pdr-1*-downregulated MK-untreated groups (Figure 7B,C). In the NL5901 strain, compared with the control RNAi group, *pdr-1* RNAi decreased the expression of *pdr-1* by 76% (No. 2, *p* < 0.001) (Figure 7D). *Pdr-1*-downregulated MK-treated NL5901 worms did not show a lessening of YFP fluorescence intensity related to α-synuclein accumulation compared with the *pdr-1*-downregulated MK-untreated group (Figure 7E,F). Based on the Western blotting analysis, *pdr-1*-downregulated MK-treated NL5901 worms did not show a decline in the α-synuclein protein level compared with the *pdr-1*-downregulated MK-untreated group (Figure 7G).

### 2.9. Parkin siRNA Reversed Maackiain-Mediated Anti-Apoptosis in the 6-OHDA-Exposed SH-SY5Y Cell Line

**Figure 8:**
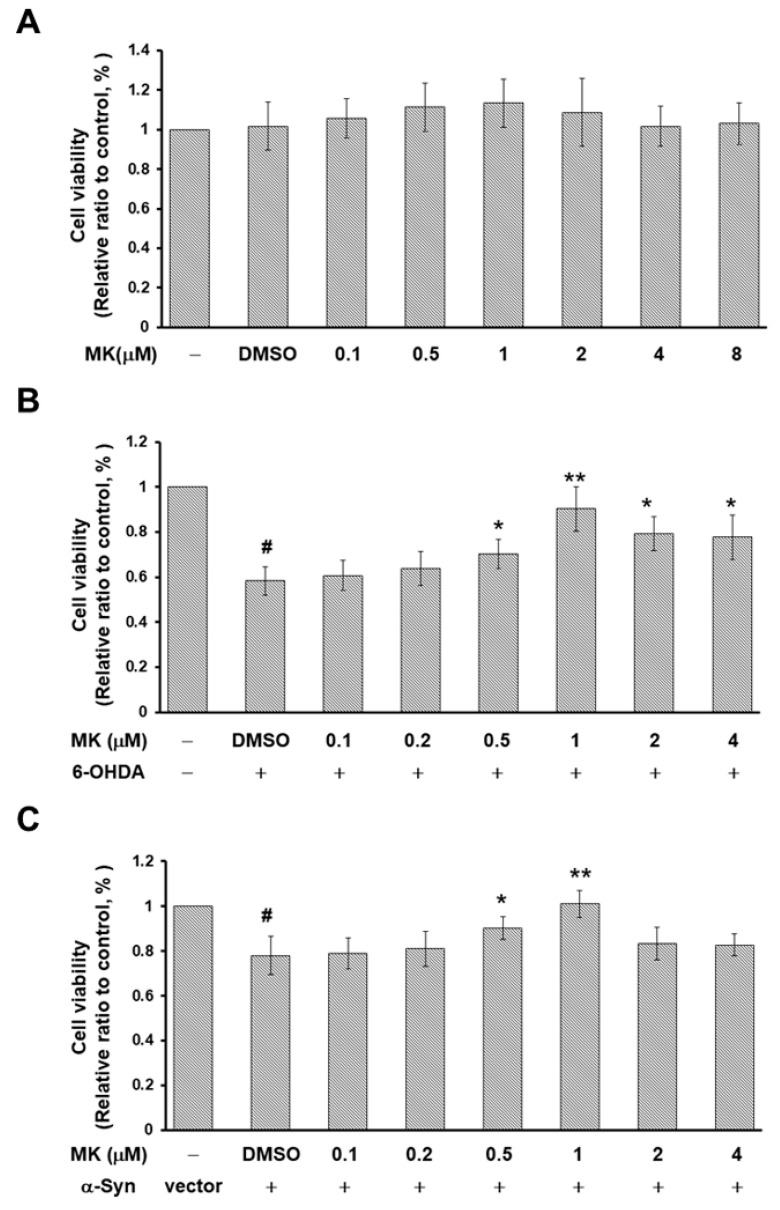
Protective effect of maackiain (MK) against 6-hydroxydopamine (6-OHDA) and α-synuclein (α-Syn)-induced toxicity in SH-SY5Y cell line. (**A**) Cell viability was measured by the 3-(4,5-dimethylthiazol-2-yl)2,5-diphenyltetrazolim bromide (MTT) assay. Cells were pretreated with 0.1, 0.5, 1, 2, 4, or 8 μM MK for 24 h. (**B**) Cell viability was measured by MTT assay. Cells were pretreated with 0.1, 0.2, 0.5, 1, 2, or 4 μM MK for 24 h and were then exposed with 100 μM 6-OHDA for an additional 24 h. # shows significant differences between 6-OHDA-exposed and control cells (# *p* < 0.001); * shows significant differences between the MK-pretreated 6-OHDA-exposed and MK-untreated 6-OHDA-exposed cells (* *p* < 0.05, ** *p* < 0.01). (**C**) α-Syn-overexpressing cell viability was measured by MTT assay. α-Syn-overexpressing cells were pretreated with 0.1, 0.2, 0.5, 1, 2, or 4 μM MK for 24 h. # shows significant differences between α-Syn-overexpressing and control cells (# *p* < 0.01); * shows significant differences between the α-Syn-overexpressing MK-pretreated and α-Syn-overexpressing MK-untreated groups (* *p* < 0.05, ** *p* < 0.01).

**Figure 9:**
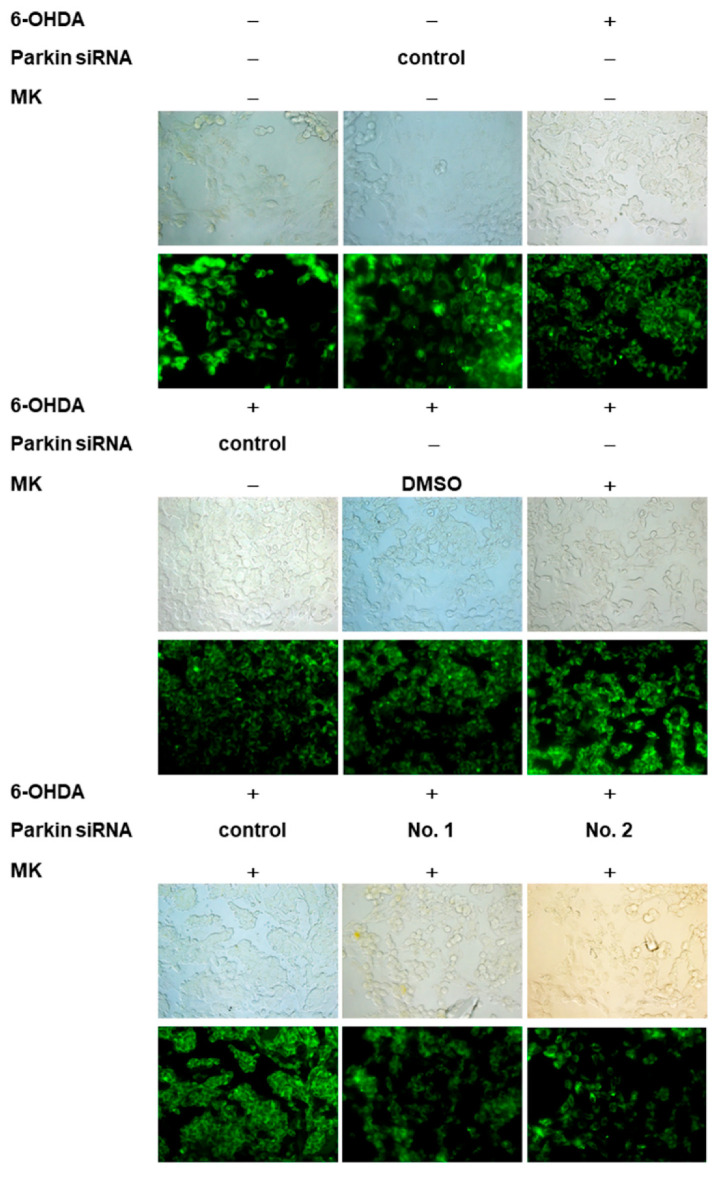

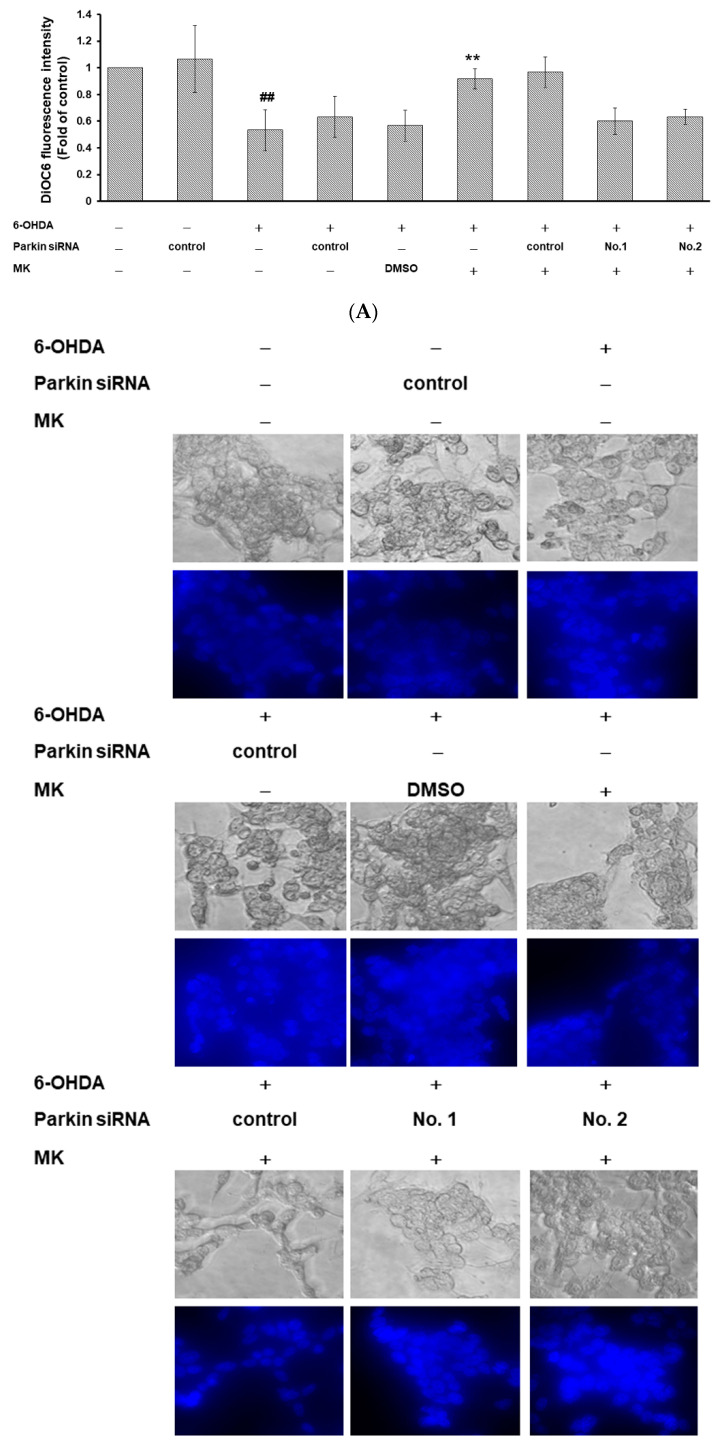

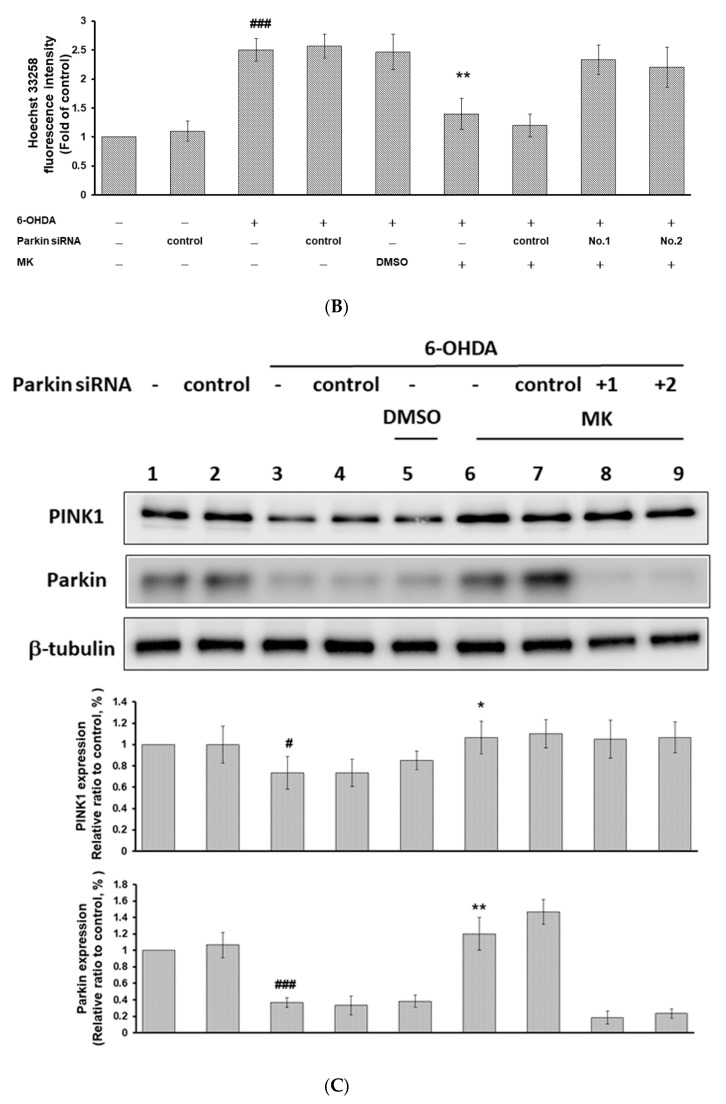
Parkin-siRNA reversed the anti-apoptotic effect of maackiain (MK) in the 6-hydroxydopamine (6-OHDA)-exposed SH-SY5Y cell line. SH-SY5Y cells were transfected with control siRNA or parkin-siRNA for 24 h. Transfected cells were incubated with 1 μM MK for 24 h and then exposed to 100 μM 6-OHDA for 18 h. (**A**) The loss in mitochondrial membrane potential (MMP) was observed by using DiOC6 dye (1 μM). Top, representative phase contrast images and fluorescent images; bottom, the fluorescence intensity of DiOC6 was analyzed by using ImageJ. The relative fold fluorescence intensity is represented as the ratio relative to the control experiment. (**B**) Nuclear condensation was observed by using Hoechst 33258 staining. Top, representative phase contrast images and fluorescent images; bottom, the fluorescence intensity of Hoechst 33258 (5 μg/mL) was analyzed by using ImageJ. The relative fold fluorescence intensity is represented as the ratio relative to the control experiment. (**C**) Western blotting analysis was used to quantify the protein level of PINK1 and parkin. One representative result is shown. The expression of β-tubulin was used as an internal control. The relative fold protein level is represented as the ratio relative to the control experiment. In the above experiments, # shows significant differences between 6-OHDA-exposed and control groups (# *p* < 0.05, ## *p* < 0.01, ### *p* < 0.001); * shows significant differences between the MK-pretreated 6-OHDA-exposed and MK-untreated 6-OHDA-exposed groups (* *p* < 0.05, ** *p* < 0.01).

To further confirm the efficacy of MK in improving PD, we used a 6-OHDA-exposed and α-synuclein-overexpressing human SH-SY5Y cell line model. According to the results of the 3-(4,5-Dimethylthiazol-2-yl)-2,5-diphenyltetrazolium bromide (MTT) assay, MK at concentrations up to 8 μM was not toxic to SH-SY5Y cells (Figure 8A). When the MK concentration is 1 μM, the viability of cells exposed to 6-OHDA and overexpressing α-synuclein can increase by 1.6-fold (*p* < 0.01) and 1.3-fold (*p* < 0.01), respectively (Figure 8B,C). Next, we wanted to evaluate the effects of MK in the apoptosis induced by 6-OHDA. Cells treated with 6-OHDA showed reduced mitochondrial membrane potential by 47% (*p* < 0.01) and increased nuclear condensation by 2.5-fold (*p* < 0.001) compared with 6-OHDA-untreated cells (Figure 9A,B). Western blotting analysis showed that protein expression of PINK1 and parkin were decreased by 27% (*p* < 0.05) and 63% (*p* < 0.001), respectively, in 6-OHDA-exposed cells compared with unexposed cells (Figure 9C). However, MK pretreatment could ameliorate these 6-OHDA-induced events. Mitochondrial membrane potential increased by 1.8-fold (*p* < 0.01) and nuclear condensation was decreased by 44% (*p* < 0.01) in MK (1 μM)-pretreated 6-OHDA-exposed cells compared with MK-untreated 6-OHDA-exposed cells (Figure 9A,B). Western blotting analysis showed that protein levels of PINK1 and parkin were increased by 1.5-fold (*p* < 0.05) and 3.2-fold (*p* < 0.01), respectively, in MK-pretreated 6-OHDA-exposed cells compared with MK-untreated 6-OHDA-exposed cells (Figure 9C). After cells were transfected with parkin siRNA, the capacity of MK to reverse the 6-OHDA-induced apoptosis was suppressed.

### 2.10. Parkin siRNA Obstructed Maackiain-Mediated Enhancing of Ubiquitin-Proteasome System and Induction of Autophagy in an α-Synuclein-Overexpressing SH-SY5Y Cell Line

**Figure 10:**
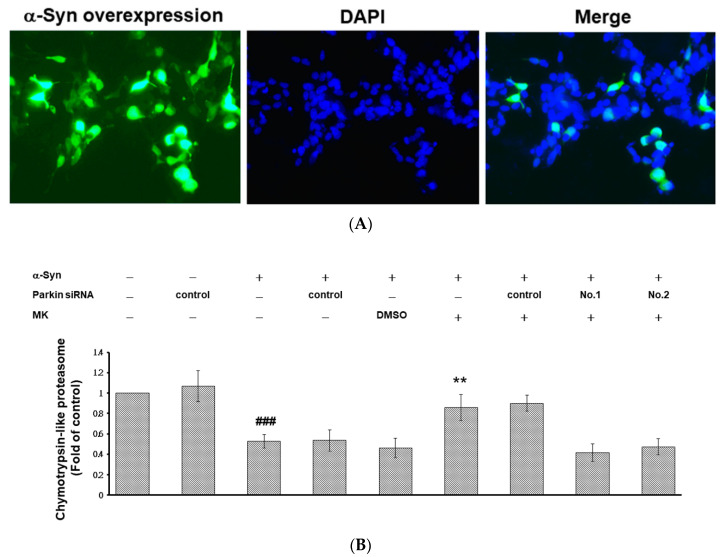

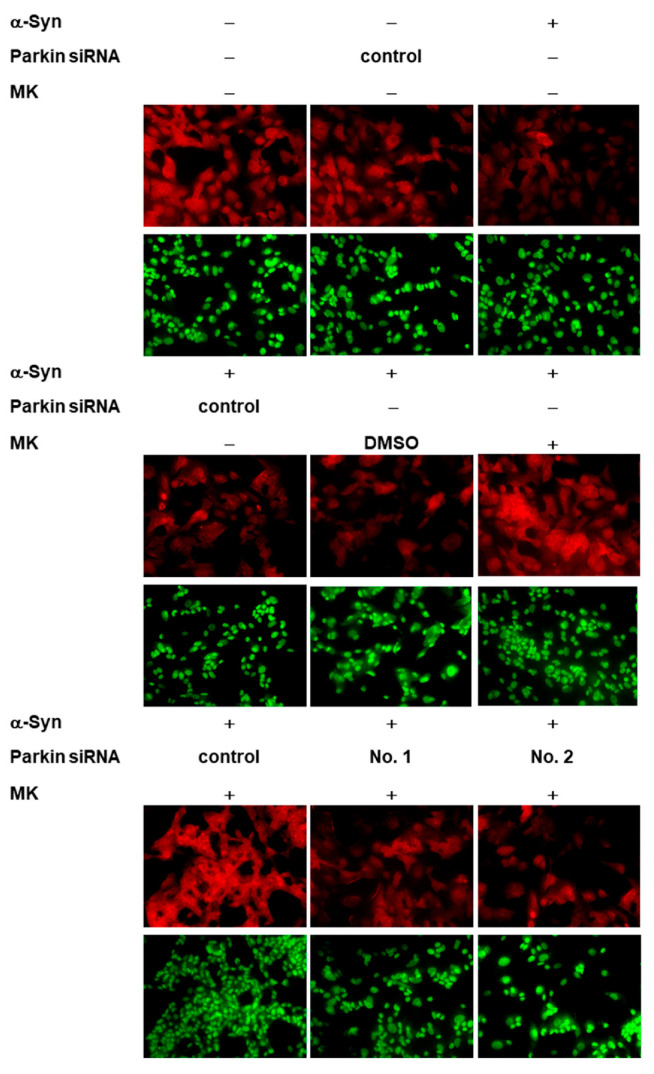

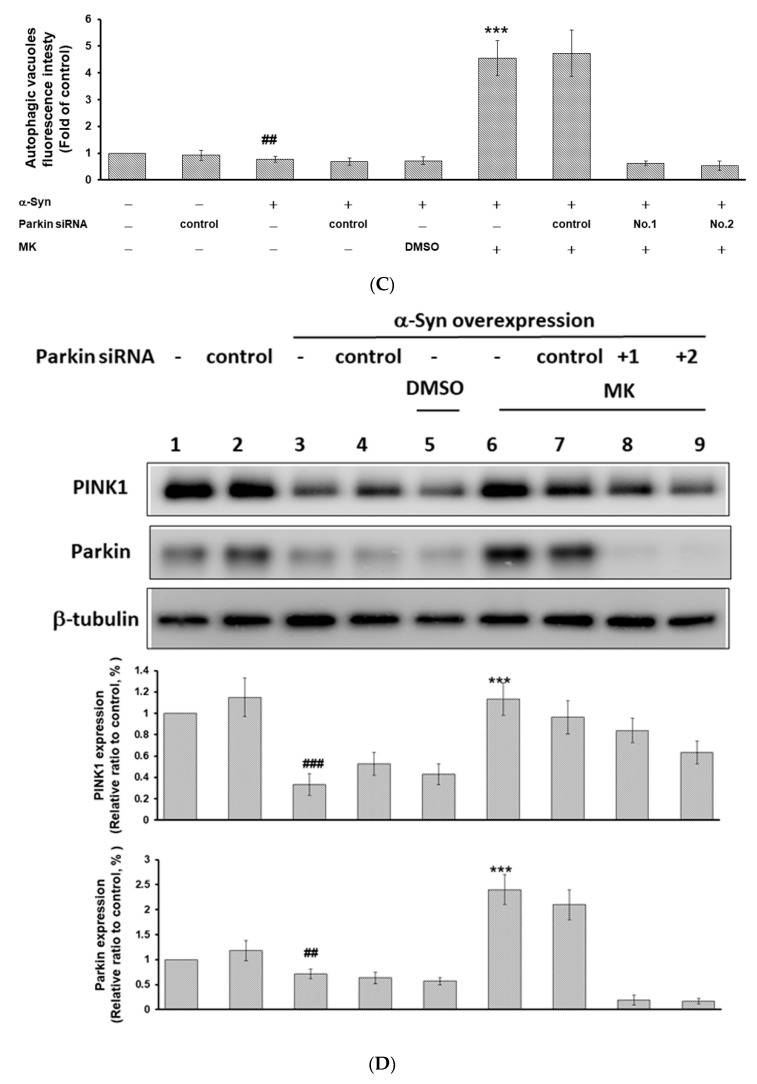
Parkin-siRNA reversed the clearance ability of maackiain (MK) in the α-synuclein (α-Syn)-overexpressing SH-SY5Y cell line. α-Syn-overexpressing SH-SY5Y cells were transfected with control siRNA or parkin-siRNA for 24 h. Transfected Cells were incubated with 1 μM MK for 24 h. (**A**) A representative fluorescence image of α-Syn-overexpressing SH-SY5Y Cells. Recombinant α-Syn was detected by Myc antibody (Green). Hoechst 33258 was used as a marker of nuclear morphology (blue). (**B**) Suc-Leu-Leu-Val-Tyr-AMC was used as a substrate to measure proteasome activity. The relative fold proteasome activity is represented as the ratio relative to the control experiment. (**C**) Autophagic vacuoles were observed by using acidic vesicular organelle (AVO) staining. Top, representative phase contrast images and fluorescent images; bottom, the fluorescence intensity of acridine orange (0.5 μg/mL) was analyzed by using ImageJ. The relative fold fluorescence intensity is represented as the ratio relative to the control experiment. (**D**) Western blotting analysis was used to quantify the protein level of PINK1 and parkin. One representative result is shown. The expression of β-tubulin was used as an internal control. The relative fold protein level is represented as the ratio relative to the control experiment. In the above experiments, # shows significant differences between α-Syn-overexpressing and control (## *p* < 0.01, ### *p* < 0.001); * shows significant differences between the MK-treated α-Syn-overexpressing and MK-untreated α-Syn-overexpressing experiments (** *p* < 0.01, *** *p* < 0.001).

We constructed and transfected pcDNA 3.1-*SNCA*-Myc plasmid into the SH-SY5Y cell line to acquire a cell model transiently overexpressing α-synuclein (Figure 10A). The results showed that UPS activity and autophagy were decreased by 47% (*p* < 0.001) and 23% (*p* < 0.01), respectively, in α-synuclein-overexpressing cells compared with control cells (Figure 10B,C). Western blotting analysis showed that protein expression of PINK1 and parkin were decreased by 67% (*p* < 0.001) and 29% (*p* < 0.01), respectively, in α-synuclein-overexpressing cells compared with control cells (Figure 10D). Conversely, MK treatment would improve these cellular function defects induced by α-synuclein overexpression in SH-SY5Y cells. We found that UPS activity and autophagy increased by 1.6-fold (*p* < 0.01) and 5.9-fold (*p* < 0.001), respectively, in α-synuclein-overexpressing MK-treated cells compared with α-synuclein-overexpressing MK-untreated cells (Figure 10B,C). Western blotting analysis showed that protein levels of PINK1 and parkin were increased by 3.4-fold (*p* < 0.001) and 3.4-fold (*p* < 0.001), respectively, in α-synuclein-overexpressing MK-treated cells compared with α-synuclein-overexpressing MK-untreated cells (Figure 10D). After cells were transfected with parkin siRNA, the capacity of MK to reverse the α-synuclein overexpression induced dysfunction of the UPS and autophagy was suppressed.

## 3. Discussion

MK is known for its multiple biological activities [34]. In this study, we used pharmacologic and transgenic *C. elegans* models to confirm that treatment with MK can ameliorate the degeneration of dopaminergic neurons induced by 6-OHDA, thus recovering the food-sensing behavior and life-span of worms as well as improving human α-synuclein protein accumulation in a dose-dependent manner. This is the first evidence that MK has antiparkinsonian effects in an experimental PD animal model.

The neuroprotective roles of MK in 6-OHDA-induced dopaminergic neuron degeneration model of *C. elegans* may be related to its antioxidant, and anti-apoptotic activity. In the present study, we found that MK reduced ROS levels, elevated *pink-1* and *pdr-1* expression, and then lessened 6-OHDA-induced degeneration of dopaminergic neurons. Moreover, in a human SH-SY5Y cell line model, MK reduced nuclear condensation, increased mitochondrial membrane potential by promoting the expression of PINK1 and parkin, and finally arrested 6-OHDA-induced apoptosis. We also confirmed that *parkin* expression was silenced by use of siRNA, which significantly reduced the ability of MK to inhibit apoptosis in worms and cells in 6-OHDA-exposed models. These results suggest that MK increases the expression of *parkin* to protect dopaminergic neurons from 6-OHDA-induced apoptosis.

*C. elegans*’s *Pink-1* is an ortholog of human PINK1 that controls the function, quality and morphology of mitochondria as well as cell survival [14]. Several lines of evidence illustrate that PINK1 is related to the anti-apoptotic activity of dopaminergic neurons. Down-regulation of PINK1 by RNAi augments the neuronal toxicity induced by 1-methyl-4-phenyl-1,2,3,6-tetrahydropyridine (MPTP) or rotenone and decreases mitochondrial membrane potential, ATP synthesis, and survival in SH-SY5Y cells [35,36]. In PINK1-deficient mice, dopaminergic neurons in the substantia nigra pars compacta are more sensitive to MPTP-induced neuronal degeneration [37]. Moreover, staurosporine-induced apoptosis in SH-SY5Y cells can be reduced by overexpression of PINK1 [38]. Overexpression of PINK1 can suppressed the loss of mitochondrial membrane potential and the apoptotic neuronal death induced by the proteasome inhibitor MG132, and the mechanism of these effects is believed to be via blocking the release of mitochondrial cytochrome *c* and activation of caspase-3 [39,40].

*Pdr-1* of *C. elegans* is an ortholog of human parkin that can bind to ubiquitin-conjugating enzyme [41]. Parkin has been shown to be inactivated in brains of PD patients and in PD animal models [42]. Accumulating evidence shows that parkin possesses neuroprotective effects by preventing neurotoxin-induced oxidative stress [43]. Treatment of human SH-SY5Y cells with 6-OHDA, rotenone, and dopamine decreases the activity of parkin, which leads to increased cell death [44,45]. Yang et al. indicated that inhibition of *parkin*’s expression makes PC12 cells more likely to die from treatment with ROS or the proteasome inhibitor lactacystin [46]. In addition, the down-regulation of *parkin* in SH-SY5Y cells blocks the ability of the endoplasmic reticulum stress inhibitor salubrinal to improve rotenone-exposed cytotoxicity [47]. Furthermore, overexpression of parkin protects SH-SY5Y cells against the ROS stress and apoptosis related to caspase 3 activation induced by 6-OHDA or manganese and is associated with enhanced survival of dopaminergic neurons [48,49]. In mouse PD models, overexpression of parkin prevents MPTP-induced degeneration of dopaminergic neurons and motor dysfunction [50]. In the nigrostriatal dopamine system of rats, overexpression of parkin can improve methamphetamine-induced neurotoxicity and tyrosine hydroxylase reduction in the substantia nigra pars compacta and striatum [51]. Dai et al. also showed that the overexpression of parkin significantly reduces excessive ROS generation, mitochondrial fragmentation, mitochondrial depolarization, and cell apoptosis in SH-SY5Y cells caused by treatment with pesticide chlorpyrifos [52]. Lin et al. showed that the neuroprotective role of parkin is related to the promotion mitochondrial fusion and inhibition of the release of cytochrome *c* by upregulation of IκB kinase (IKK)/nuclear factor-κB (NF-κB)/optic atrophy 1 (OPA1) axis [8,53]. Lin et al. also indicated that 6-OHDA-induced apoptosis is blocked by enhancing autophagy via parkin and Beclin1 interaction [10]. Therefore, the MK-mediated PINK1/parkin pathway can protect neurons against neurotoxin-induced toxicity in vitro and in vivo.

Parkin expression induced by bioactive compounds may improve neurotoxin-induced apoptosis and movement dysfunction in PD models. For example, Khasnavis et al. indicated that oral administration of cinnamon can improve the level of parkin and the loss of dopaminergic neurons in the substantia nigra of MPTP-injected mice [54]. Sonia Angeline et al. showed that naringenin or sesamol can improve the reduction in parkin expression and impairment of motor function in a rotenone-exposed rat PD model [55]. In this study, MK effectively reversed the 6-OHDA-induced inhibition of the expression of PINK1 and parkin to decrease dopaminergic neurodegeneration, an effect that was shown previously for carnosic acid [9], salidroside [56], schizandrin A [57], and *Ganoderma lucidum* extract [58].

Previous research showed that PD is linked with the accumulation of intracellular inclusions known as Lewy bodies under stressful conditions and lessened UPS activity [59] and autophagy function [60]. Thus, augmenting the UPS or autophagy with the resultant degradation of misfolded and aggregated proteins may play a key role in prevention PD. In the present study, we found that MK increased UPS activity and autophagy by upregulating *pink1*/*pdr-1* (*parkin*) expression and therefore reduced α-synuclein accumulation in the *C. elegans* and SH-SY5Y cell line models. When *parkin* expression was silenced by use of siRNA, the ability of MK to arrest the effects of α-synuclein accumulation was significantly abolished in both models. The critical role of parkin in neuroprotection is proved by the fact that 26S proteasome activity is blocked in parkin-knockdown mice [21]. Clinical studies have shown that some PD patients in their brains have *parkin* mutations and involved Lewy bodies [61]. Inhibition of the mouse *parkin* gene reduces degradation of α-synuclein aggregates and causes death of dopaminergic neurons [62]. Lonskaya et al. indicated that parkin promotes autophagosome maturation by interacting with Beclin1 and then degrades α-synuclein in transgenic SNCA (A53T) mice [63]. The use of the tyrosine kinase inhibitors nilotinib and bosutinib in those mice can promote parkin interaction with Beclin1 and stimulate α-synuclein clearance to protect dopaminergic neurons [19]. Thus, MK regulates PINK1/parkin axis to promote the UPS and autophagy activities and to avoid α-synuclein accumulation in vitro and in vivo. Some bioactive compounds can also act to decrease the accumulation or aggregation of α-synuclein. For example, caffeic acid can activate JNK/Bcl-2-mediated autophagy to decrease A53T α-synuclein in the SH-SY5Y cell line [64]. Ginkgolic acid can stimulate autophagy to clear α-synuclein aggregates [65].

Monoamine oxidase (MAO) catalyzes the oxidation of monoamines, and its two isoforms, MAO-A and MAO-B, break down neurotransmitter dopamine. MK was observed as a new selective, and reversible, MAO-B inhibitor with the potential to relieve symptoms of PD [27]. Moreover, pro-inflammatory mediators cause greater damage to dopaminergic neurons than to other cell types [66]. In a previous report, MK exhibited inhibitory effects on LPS-induced nitric oxide production in RAW 264.7 macrophages [30] and inhibited 5-lipoxygenase and OVA-induced airway inflammation [67]. Moreover, Singh et al. showed that parkin can target the nucleotide binding oligomerization domain containing 2 protein (NOD2) to regulate astrocyte endoplasmic reticulum stress and inflammation [68]. In our study, MK could increase parkin expression and may reverse astrocyte-associated inflammation. Therefore, MK may lessen dopaminergic neuron damage by improving the chronic inflammatory niche in the brain of PD patients. Some large-scale cohort studies have found that metabolic-related diseases such as diabetes can increase the risk of PD [69]. Huang et al. reported that MK has antidiabetic activity via regulation of AMP-activated protein kinase activity [34]. Therefore, MK may also decrease the risk of PD by reducing the incidence of diabetes.

In summary, our experimental data showing improved 6-OHDA-induced and α-synuclein-accumulated neurotoxicity in PD models confirm that MK may have considerable therapeutic applications for PD. MK enhances anti-apoptosis, the UPS, and autophagy by augmenting the PINK1/parkin pathway.

## 4. Materials and Methods

### 4.1. Chemicals, C. elegans Strains, and Worm Synchronization

Synthesized MK (mol. wt. 284.27, 98% purity) was purchased from Rainbow Biotechnology Co. Ltd. (Shilin, Taipei, Taiwan) and dissolved in DMSO as a stock solution (1 M). Other chemicals and culture media were purchased from Sigma-Aldrich (St. Louis, MO, USA) unless otherwise stated. Wild-type Bristol N2 *C. elegans*, transgenic BZ555 strain (Pdat-1:: GFP), transgenic N5901 strain (Punc-54::α-Syn::YFP), transgenic DA2123 strain (Plgg-1:: GFP), and *E. coli* strain OP50 were acquired from the Caenorhabditis Genetics Center (University of Minnesota, Saint Paul, MN, USA). Maintenance and synchronization of *C. elegans* were conducted by using a previously described method [70].

### 4.2. Food Clearance Test

A food clearance test of *C. elegans* was carried out according to a previously described method [71,72] to determine the suitable concentration of MK for treatment. *E. coli* OP50 grew overnight and were then resuspended at a final optical density (OD) of 6.6 in nematode S-medium. MK was diluted into the *E. coli* suspension, in order to achieve the desired concentrations. Each well of a 96-well plate received 50 µL of the *E. coli* suspension. Approximately 20 synchronized L1 worms in 10 µL of S-medium were added to an *E. coli* suspension containing a series of MK concentrations, and were then incubated in a 96-well microtiter plate at 20 °C. Plates were covered with sealers to prevent evaporation. The optical density of the culture at 595 nm was measured once/day (d) for 6 d, using a SpectraMax M2 Microplate Reader (Molecular Devices, Silicon Valley, CA, USA). Before measuring OD_595_, each plate was placed onto a plate shaker for 30 min. The fraction of worms alive per well was observed microscopically on the basis of size.

### 4.3. 6-OHDA-Induced Dopaminergic Neuron Degeneration and Maackiain Treatment

For 6-OHDA-induced dopaminergic neuron degeneration in *C. elegans*, we performed the previous protocol with slight modifications [70]. First, L1 worms were transferred to OP50/NGM plates with or without MK at 20 °C for 65 h (L3 stage) and were then exposed to 6-OHDA (50 mM) for 1 h. After exposure, worms were washed with M9 buffer and transferred to OP50/NGM/5-fluoro-2′-deoxyuridine,2′-deoxy-5-fluorouridine (FUDR, 0.04 mg/mL) plates and cultured for 3 days at 20 °C for various assays.

### 4.4. Analysis of Dopaminergic Neuron Degeneration 

The analysis of dopaminergic neuron degeneration of worms was done by the previous described the fluorescence quantitative method [73]. After 3 d of treatment at 20 °C, worms were washed three times with M9 buffer and then mounted onto a 2% agar pad on a glass slide, anesthetized with 100 mM sodium azide, before being enclosed with a coverslip. Imaging of the head region of the immobilized worms was visualized with a Zeiss Axio *Imager A1* fluorescence microscope (Carl Zeiss MicroImaging GmbH, Göttingen, Germany). Fluorescence intensity was determined using ImageJ software (National Institutes of Health, Bethesda, MD, USA). Loss of the GFP signal from DA neurons indicated DA neuron degeneration. Moreover, if any part of the dendrites of the dopaminergic neuron of the worm showed bubbles or was absent when observed with a stereomicroscope, the worm was considered positive for dopaminergic neuron neurodegeneration.

### 4.5. Food-Sensing Behavior Test

We used the food-sensing behavior test to measure the function of dopaminergic neurons in worms by a previously described protocol [74]. Briefly, assay plates were prepared by spreading *E. coli* in a ring with an inner diameter of 1 cm and an outer diameter of 8 cm, and incubated overnight at 37 °C on 9-cm diameter NGM agar plates to prevent the worms from reaching the edge of the plate during the assay. Well-fed 6-OHDA-treated or MK/6-OHDA-treated N2 adult worms (After 3 d of treatment at 20 °C) were washed with M9 buffer and then transferred in a drop of M9 buffer to the center of an assay plate. Five min after transfer (settle down), the locomotory rate of each worm on the empty lawn and the bacterial lawn was measured three times at 20 s intervals/time, respectively. The slowing rate was calculated as the percentage of the locomotory rate in the bacterial lawn compared to the rate in the absence of a bacteria lawn. In all analyses, plates were anonymously labeled so that the experimenter was blind to worm treatment. Then the food-sensing behavior of each group was compared with the control. The average slowing rate of 50 worms was calculated for each group.

### 4.6. Life-Span Test

Life-span tests were performed using a previously described protocol [75]. The test plates were prepared by adding MK stock solution, at various concentrations, to NGM plates just before use. The NGM plates were then seeded with OP50. Life-span analyses were performed by transferring control, 6-OHDA-treated, and MK/6-OHDA-treated L3 stage worms to a new plate every 3 d, until all worms were dead. A total of 0.04 mg/mL of FUDR was added to each plate to reduce progeny production. Survival was calculated daily, and the worms were counted as dead if they failed to respond to mild, repeated touches with a platinum pick. Age 1 d was defined as the first day of adulthood. Worms that moved off the walls of the plates and died from dehydration were excluded from the analyses. Survival curves are shown using the product-limit method of Kaplan and Meier by use of SPSS software (IBM, Armonk, NY, USA).

### 4.7. Analysis of α-Synuclein Accumulation

Accumulation of α-synuclein protein was measured by using a previously described quantitative fluorescence protocol [73]. Synchronized NL5901 L3 larvae were cultured on OP50/NGM plates containing 0.04 mg/mL FUDR, with or without the indicated concentrations of MK, for 3 d at 20 °C, and then washed three times with M9 buffer. Worms were examined using a fluorescence microscope as described in the dopaminergic neurodegeneration assay, in order to monitor the YFP signal (the accumulation of α-synuclein protein) for the head region of each worm. The signal was quantified by measuring fluorescence intensity using ImageJ software (National Institutes of Health).

### 4.8. Analysis of Protein Expression of C. elegans

We used Fastprep24 (MP Biomedicals LLC, Solon, OH, USA) with protease inhibitors/PBS to obtain protein extracts from frozen whole-worm pellets. Western blotting was performed by using a previously described protocol [76]. Samples were boiled 10 min with sodium dodecyl sulfate (SDS) sample buffer, and separated on 10% sodium dodecyl sulfate polyacrylamide gel electrophoresis (SDS-PAGE). Then proteins were transferred to polyvinylidene difluoride (PVDF) membranes. Antibody binding was visualized by binding of horse-radish peroxidase (HRP)-coupled secondary antibody and Amersham enhanced chemiluminescence system (Amersham Biosciences, Piscataway, NJ, USA). Signals were detected by using a BioSpectrum Imaging System (UVP, Upland, CA, USA) Human α-synuclein monoclonal antibody and β-actin antibody were purchased from Santa Cruz Biotechnology (Santa Cruz, CA, USA).

### 4.9. Reactive Oxygen Species Assay

We used a previously described protocol to perform the 2′,7′-dichlorodihydrofluorescein diacetate (H_2_DCFDA) assay [77] for worms with a SpectraMax M2 Microplate Reader (Molecular Devices, Silicon Valley, CA, USA). Briefly, Thirty N2, 6-OHDA-treated or MK/6-OHDA-treated worms, on day 3, were washed with M9 buffer 3 times and transferred to a 96-well plate with 150 μL of PBS per well. Subsequently 50 μL of H_2_DCFDA (150 μM) dissolved in PBS buffer was added and immediately fluorescence was measured for 150 min at 15 min intervals at 20 °C, using excitation and emission λ at 485 nm and 520 nm (SpectraMax M2 Microplate Reader, Molecular Devices, Silicon Valley, CA, USA), respectively.

### 4.10. RNA Extraction and qPCR of C. elegans

We used TRIzol reagent (Invitrogen, Carlsbad, CA, USA) with glass beads to extract total RNA from worms. For qPCR analyses, we used the SuperScript One-Step RT-PCR kit (Invitrogen), SYBR Green I Master kit (Roche Diagnostics, Indianapolis, IN, USA), and an ABI StepOnePlus system (Applied Biosystems, Inc., Foster City, CA, USA) according to the manufacturer’s instructions. Primer pairs are listed in Table 1. Fold-differences were calculated using the comparative 2ΔΔ*C*_t_ method and *β-actin* expression as an endogenous control.

### 4.11. Proteasome Activity Assays of C. elegans

Proteasome activity (chymotrypsin-like activity) was measured in worms using a previously described method [76]. Briefly, using a Precellys 24 homogenizer, worms were lysed in a proteasome activity assay buffer containing 50 mM Tris-HCl (pH = 7.5), 250 mM sucrose, 2 mM adenosine triphosphate (ATP), 5 mM MgCl_2_, 1 mM dithiothreitol, and 0.5 mM ethylenediaminetetraacetic acid (EDTA). The lysate was centrifuged at 10,000× *g* for 15 min at 4 °C. For each test, 25 μg of total lysate was loaded into each well of a 96-well microtiter plate, after which fluorogenic substrate was added. Suc-Leu-Leu-Val-Tyr-AMC (Sigma-Aldrich, St. Louis, MO, USA) was used as a substrate for testing the chymotrypsin-like activity of the proteasome. After incubation for 1 h at 25 °C, fluorescence (excitation wavelength = 380 nm, emission wavelength = 460 nm) was measured with a SpectraMax M2 Microplate Reader (Molecular Devices, Silicon Valley, CA, USA).

### 4.12. Autophagy Activity Assay of C. elegans

We used a previously described protocol to perform an assay of autophagy activity using GFP-tagged LGG-1 transgenic worms (strain DA2123) [76]. DA2123 is a transgenic worm that expresses GFP-tagged LGG-1. Synchronized DA2123 L3 larvae were cultured on OP50/NGM plates containing FUDR, with or without the indicated concentrations of MK, for 3 d at 20 °C, and then washed three times with M9 buffer. Then worms were observed using a fluorescence microscope as described in the dopaminergic neurodegeneration assays. LGG-1::GFP positive puncta regions in lateral epidermal seam cells were examined. LGG-1::GFP positive puncta regions were counted in at least 150 seam cells. At least 50 nematodes were counted per group.

### 4.13. RNA-mediated interference of C. elegans

We used a previously described method to perform the RNA-mediated interference (RNAi) of *C. elegans* [76] by feeding worms with RNase III-resistant *E. coli* expressing the *pdr-1*-specific double-stranded RNA (Open Biosystems, Huntsville, AL, USA), which caused the specific degradation of the targeted endogenous mRNA. In the 6-OHDA-exposed model, starved L1 worms were transferred every 24 h to new RNAi/MK plates and were fed until the third day for 6-OHDA exposure. In the transgenic NL5901 model, L1 worms were fed on RNAi/MK plates until the third day for the α-synuclein accumulation assay.

### 4.14. Culture of the SH-SY5Y Cell Line and Treatment with 6-OHDA 

Human neuroblastoma SH-SY5Y cells (passage 20) were a generous gift from Chia-Wen Tsai (China Medical University, Taichung, Taiwan). Cell culture methods refer to previously published literature [9]. DMEM, penicillin-streptomycin, trypsin-EDTA, and fetal bovine serum were purchased from Gibco, ThermoFisher Scientific (Waltham, MA, USA). Cells were plated on 35 mm dishes (BD Falcon, NY, USA) at a density of 1.0 × 10^6^ cells per dish, and then incubated with 1 μM MK for 24 h and then exposed to 100 μM 6-OHDA for 18 h.

### 4.15. Generation of an SH-SY5Y Cell Line Transiently Overexpressing α-Synuclein

To obtain an SH-SY5Y cell line overexpressing *α*-synuclein, the synthetic coding sequence of *SNCA* (Accession numbers: NM_000345, Genewiz Inc., South Plainfield, NJ, USA) was amplified and cloned into the pcDNA3.1(+)-Myc vector (Invitrogen, ThermoFisher Scientific, Carlsbad, CA, USA) using the restriction enzymes *Nhe*I and *Apa*I (New England Biolabs, Beverly, MA, USA). Then pcDNA3.1 (+)-*SNCA*-Myc or pcDNA3.1 (+)-control vectors were transfected to the SH-SY5Y cell line by Lipofectamine 2000 reagent according to the manufacturer’s instructions. (Invitrogen, ThermoFisher Scientific, Carlsbad, CA, USA) and were selected by G418 (1.5 mg/mL).

### 4.16. Small RNA Interference of the SH-SY5Y Cell Line

The experimental method is described according to previous studies [9]. The sequence of small RNA interference (siRNA) for parkin was designed as follows: 5′-UUCGCAGGUGACUUUCCUCUGGUCA-3′ (Tri-I Biotech Inc, Taipei, Taiwan). In the 6-OHDA-exposed SH-SY5Y line, cells were transfected with control siRNA or parkin siRNA for 24 h, were pretreated with 1 μM MK for another 24 h, and finally were treated with 100 μM 6-OHDA for 18 h. In the α-synuclein overexpressing SH-SY5Y cell line, cells were transfected with control siRNA or parkin siRNA for 24 h and were then treated with 1 μM MK for another 24 h.

### 4.17. Western Blot Analysis of the SH-SY5Y Cell Line

For Western blot analysis of SH-SY5Y cells, we followed the experimental method described in the previous study [9]. Monoclonal antibodies to PINK1, parkin, and β-tubulin and HRP goat anti-rabbit secondary antibodies were purchased from Santa Cruz Biotechnology, Inc. (Santa Cruz, CA, USA).

### 4.18. Nuclear Staining of Hoechst 33,258 and Measurement of Mitochondrial Membrane Potential in the SH-SY5Y Cell Line

For nuclear staining (Hoechst 33258, 5 μg/mL) and measurement of mitochondrial membrane potential (DiOC6 dye, 1μM) in the SH-SY5Y cell line, we followed the experimental method described in the previous study [78]. Cells were washed with PBS and then were fixed with 3.7% paraformaldehyde (pH = 7.4) solution for 50 min and stained with Hoechst 33258 for 1 h at 25 °C in the dark. Morphological changes were observed using a fluorescence microscope. The fluorescence intensity of Hoechst 33258 was analyzed by using ImageJ software (National Institutes of Health). In the measurement of mitochondrial membrane potential, cells were washed with PBS and then were exposed to DiCO6 dye (1 μM) in the incubator for 30 min. The fluorescence microscope was used to detect changes in MMP. The images were quantified for fluorescence intensity using ImageJ software (National Institutes of Health).

### 4.19. Immunofluorescence Staining

For immunofluorescence staining, we followed the experimental method described in the previous study [8]. The primary Myc antibody was purchased from Santa Cruz Biotechnology, Inc. (Santa Cruz, CA, USA). The Alexa Fluor 488 goat anti-rabbit IgG secondary antibody was purchased from Invitrogen (ThermoFisher Scientific).

### 4.20. Proteasome Activity Assay and Acidic Vesicular Organelle Staining in the SH-SY5Y Cell Line

For the proteasome activity assay (chymotrypsin-like activity) and acidic vesicular organelle (AVO) staining (acridine orange, 0.5 μg/mL) in the SH-SY5Y cell line, we followed the experimental method described in the previous study [9,10]. The chymotrypsin-like activity of the proteasome was detected in cell lysates with Suc-Leu-Leu-Val-Tyr-AMC (Sigma-Aldrich, St. Louis, MO, USA) as the substrate. After incubation for 1 h at 25 °C, fluorescence was measured with a SpectraMax M2 Microplate Reader (Molecular Devices, Silicon Valley). In the AVO staining, cells were washed with PBS and incubated with acridine orange hydrochloride solution for 10 min at 37 °C in the dark. The formation of AVO was detected under the fluorescence microscope. The images were quantified for fluorescence intensity using ImageJ software (National Institutes of Health).

### 4.21. Statistical Analyses

Statistical analyses were implemented using SPSS software (SPSS, Inc., Chicago, IL, 2013). Each experiment was replicated three times. Data are presented as means ± standard deviations (SDs). The differences between two means were calculated by independent Student’s *t*-tests. Statistical significance was assumed when *p* < 0.05.

## Figures and Tables

**Table 1:** Primers for real time PCR.

Genes of *C. elegans*/Human	Primer Sequences (5′-3′)	(Start→End) Size (bp)
Lrk-1/LRRK1	Forward: TTTCAACACCCAATCTCCAAC Reverse: TGATACTCGCTTGCCACAC	(1983→2092) 110
Pdr-1/PRKN	Forward: TGCTCGTCAACCTCTGTTC Reverse: TCACTTTCTCCTTCCCATCAC	(376→601) 226
Pink-1/PINK1	Forward: GAGACGATACCGACAAACAC Reverse: GGCATTTCCTCCAAGACTAAC	(882→1158) 277
Djr-1.1/PARK7	Forward: CGGATTAGATGGAGCCGAAC Reverse: ATCAGCCCACCAGACTCTAC	(111→305) 195
Djr-1.2/PARK7	Forward: GCTTTGATCCTTTTGCCACC Reverse: CTGCCAGTTTGCTACATCC	(19→247) 229
Vps-35/VPS35	Forward: AACTCTGCTCAAAACTACTCAC Reverse: CCACAACCTTCTTCCCATTC	(1953→2146) 194
Catp-6/ATP13A3	Forward: TCACACCATACCAACCTCC Reverse: GTTTCCAAGAGTCTTCAGAACC	(3092→3336) 245
Dnj-27/DNAJC10	Forward: TCCACTTATTGCTCACATTGTC Reverse: TCCACCATCAACTCCACATC	(427→635) 209

## Abbreviations

DMSO	Dimethyl sulfoxide
GFP	green fluorescent protein
MK	Maackiain
MMP	Mitochondrial membrane potential
MPTP	Methyl-4-phenyl-1,2,3,6-tetrahydropyridine
MTT	3-(4,5-Dimethylthiazol-2-yl)-2,5-diphenyltetrazolium bromide
6-OHDA	6-Hydroxydopamine
PD	Parkinson’s disease
PINK1	Phosphatase and tension homologue (PTEN)-induced kinase 1
siRNA	small interfering RNA
α-Syn	α-synuclein
UPS	ubiquitin-proteasome system
YFP	yellow fluorescent protein
